# Concurrent Basal Cell Carcinoma Masquerading As Osteomyelitis of the Acromioclavicular Joint: A Rare Presentation

**DOI:** 10.7759/cureus.62619

**Published:** 2024-06-18

**Authors:** Abraham Kisule, Sakshi Bai

**Affiliations:** 1 Rheumatology, Henry Ford Health System, Jackson, USA; 2 Internal Medicine, Henry Ford Health System, Jackson, USA

**Keywords:** vismodegib, sunscreen, dermatology, osteomyelitis, basal cell carcinoma

## Abstract

Basal cell carcinoma (BCC) ranks as the most common form of skin cancer in the United States, and its prevalence continues to increase. Regular self-examinations of the skin can significantly enhance treatment outcomes. This report investigates a rare instance of BCC initially misdiagnosed as osteomyelitis, stemming from a longstanding wound on the patient’s left shoulder.

A 66-year-old male with a history of working in construction presented with a non-healing wound on his left shoulder, which he initially sustained from a metallic rod injury. Despite self-treatment, the wound deteriorated, revealing subcutaneous fat and producing foul-smelling drainage. Imaging suggested osteomyelitis, but the persistent and worsening nature of the wound over two years, previously concealed from his family and healthcare providers, prompted further investigation. A biopsy confirmed infiltrative BCC. The patient was referred to a tertiary care facility for comprehensive treatment, including long-term antibiotics for osteomyelitis and systemic therapy with vismodegib for BCC.

Basal cell carcinoma commonly appears as a pink or flesh-colored papule or nodule, often with surface features that aid in early identification and treatment. Yet, infiltrative BCC, like the case described here, can pose diagnostic challenges because of its subtle yet aggressive characteristics. The complex causes of BCC highlight the necessity of preventive actions, particularly for those with prolonged exposure to ultraviolet (UV) radiation. Treatment approaches primarily aim at removing the tumor and may incorporate targeted therapies for more advanced instances.

This case underscores the importance of regular skin self-examinations and prompt medical attention for lingering wounds, particularly among those at higher risk. Successfully addressing BCC demands a comprehensive strategy involving surgery, targeted chemotherapy, and preventive actions against potential future skin malignancies. Maintaining long-term surveillance is crucial for individuals with prior BCC diagnoses to detect any potential recurrence and address any enduring consequences of treatment.

## Introduction

Skin cancer can be detected through visual inspection without invasive procedures. Routine monthly self-examinations are recommended, particularly for high-risk individuals. Early detection significantly improves treatment outcomes. Basal cell carcinoma (BCC) is the most common type of skin cancer in the U.S., with increasing incidence. It responds well to localized treatments, but advanced cases may require targeted chemotherapy [1].

This case report describes a patient with an unusual BCC presentation on his left shoulder, initially misdiagnosed as osteomyelitis. After a biopsy confirmed BCC, the patient was referred for specialized care treatment that involved addressing the osteomyelitis with antibiotics and wound care before starting the cancer treatment with Erivedge. The patient is now recovering.

## Case presentation

**Figure 1:**
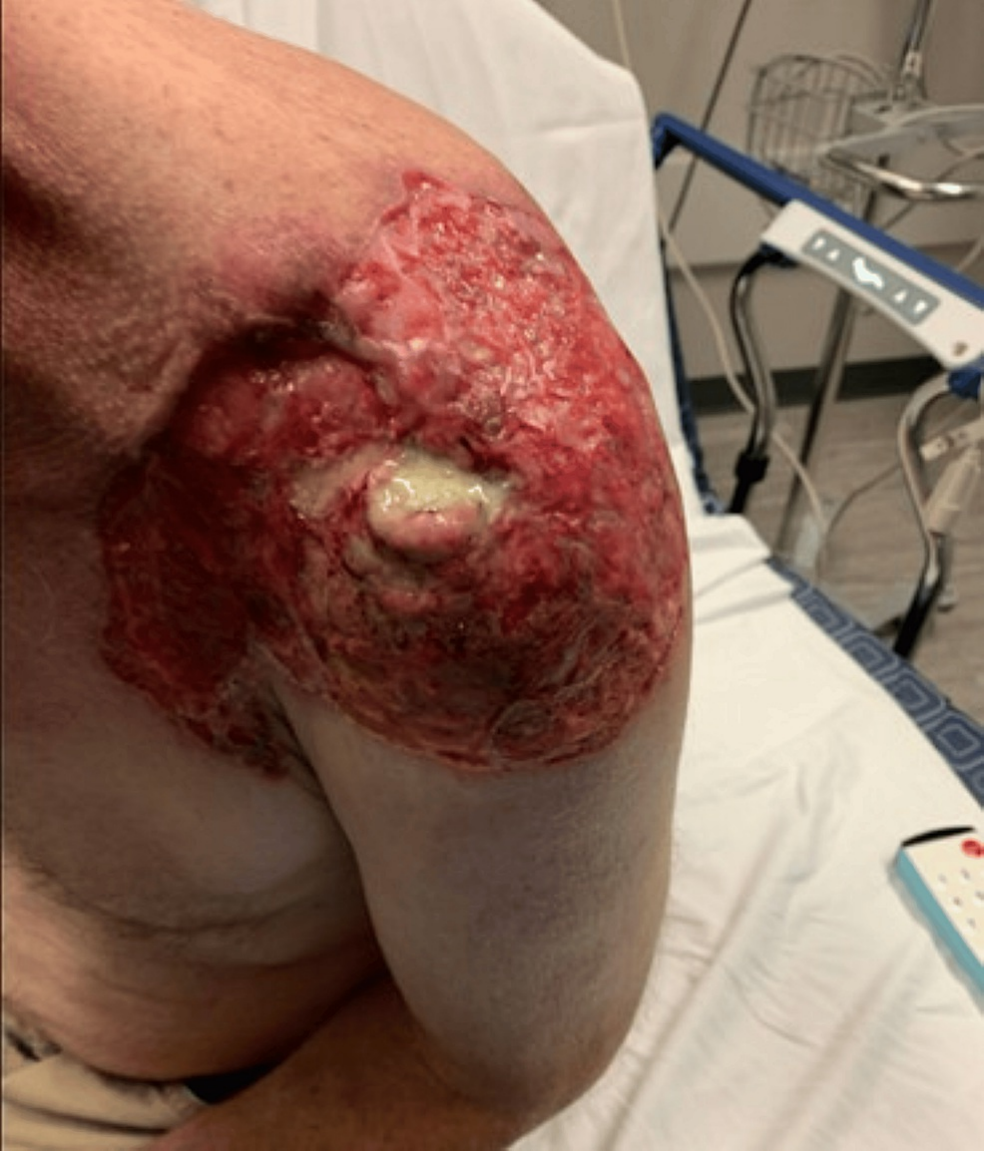
Left shoulder showing exposed left acromion

A 66-year-old male presented to our community hospital with concerns about a non-healing left shoulder wound, which he noticed had been present for two months (Figure 1).

**Figure 2:**
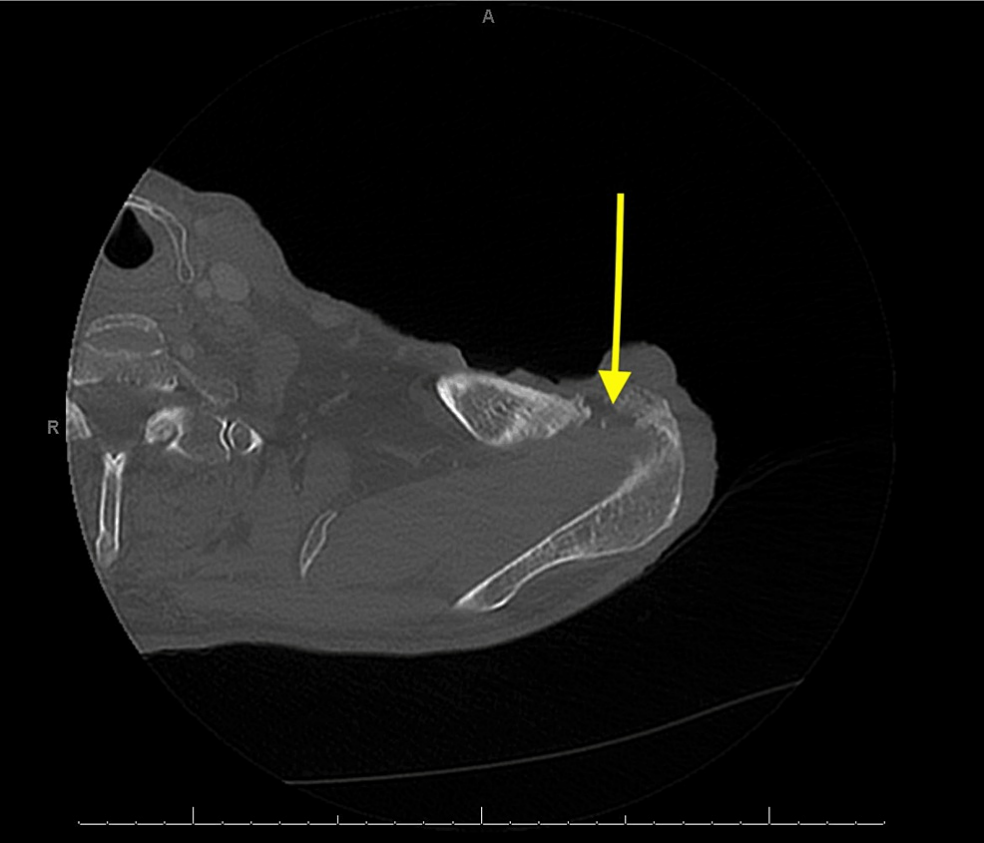
The CT scan of the left shoulder AC joint showing erosive changes (arrow) and soft tissue swelling surrounding the joint. AC: Acromioclavicular

He shared that the source of his injury was a metallic rod that impaled his shoulder while working at a construction site. He self-treated his wound with over-the-counter ointments and dressings, but unfortunately, the wound continued to worsen until it exposed the subcutaneous fat layer. He endorsed yellowish-green foul-smelling drainage from the wound site and 8/10 left shoulder pain with motion. He denied any systemic signs of infection like fevers, chills, night sweats, and body aches. A CT scan of his left shoulder showed erosive changes of the distal clavicle and acromion marginating the acromioclavicular (AC) joint, concerning septic arthritis and osteomyelitis. The CT scan also showed marked surrounding soft tissue inflammation without loculated collection or radiopaque retained foreign body (Figure 2).

**Figure 3:**
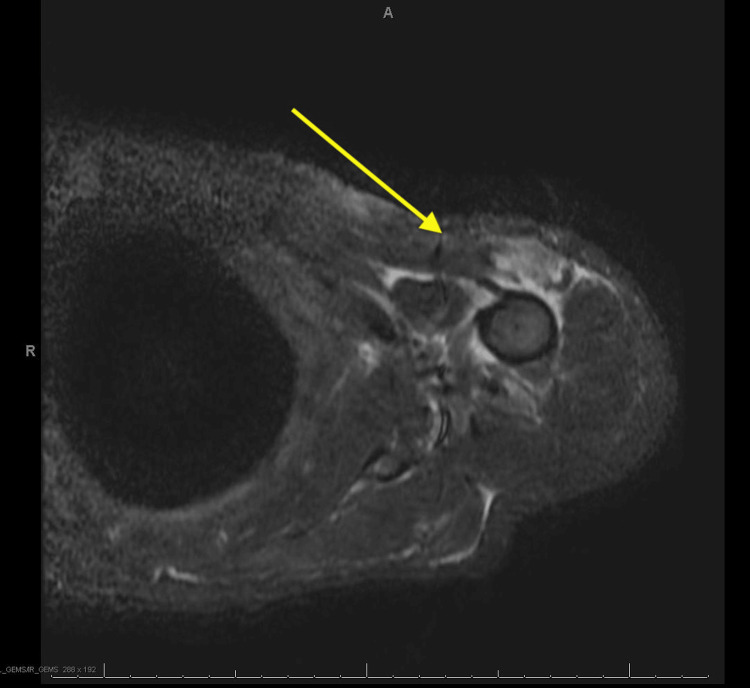
The MRI of the left shoulder showing a significant sinus tract (arrow) from the shoulder wound to the left subdeltoid bursa.

A follow-up MRI of the left shoulder was obtained to show the extent of osteomyelitis. The results of the MRI were significant for a tract from a wound to the left subdeltoid bursa (Figure 3).

On physical exam, he had a sizeable left shoulder wound that extended from his deltopectoral groove superiorly to his back, measuring approximately 25 cm. It extended laterally along his deltoid toward its insertion on the humerus, measuring approximately 15 cm. In the middle of his wound, there was a 2 cm x 3 cm area with purulent drainage with exposed bone along the posterior aspect, tracking less than 1 cm in any direction. He was treated empirically with antibiotics, vancomycin, and cefepime, pending wound culture results.

Wound cultures grew *Staphylococcus aureus* (MSSA), *Corynebacterium*, and *Streptococcus anginosus*. Antibiotics were then simplified to cefazolin alone. Given the extent of his wounds and the period during which the patient said that it had developed, it was hard to believe that osteomyelitis was the culprit driving the extent of the wound. On further questioning, the patient said he had had the wound for two years and that it had worsened over time. He had hidden this wound from his family, and since he did not follow up with a primary care physician (PCP) regularly, the wound had gone undetected for so long. With this added information, a punch skin biopsy was performed by plastic surgery. The biopsy results showed BCC, infiltrative type, with positive peripheral biopsy margins. 

The patient was then transferred to a tertiary care facility because of the need for multi-flap coverage versus free flap to be performed by advanced plastic surgery. At the tertiary center, he was continued on antibiotics for the treatment of osteomyelitis. There was no need for any immediate surgical interventions, as he required a long course of treatment with antibiotics to allow for the resolution of his osteomyelitis. In addition, adequate time was needed for his extensive wound to heal. He was discharged on amoxicillin-clavulanate 875 mg to 125 mg twice per day for six weeks and was scheduled for outpatient follow-up with a wound care clinic. Regarding his BCC, his case was discussed at the tumor board, and it was unanimously agreed upon to begin systemic treatment with vismodegib 150 mg daily. 

## Discussion

**Figure 4:**
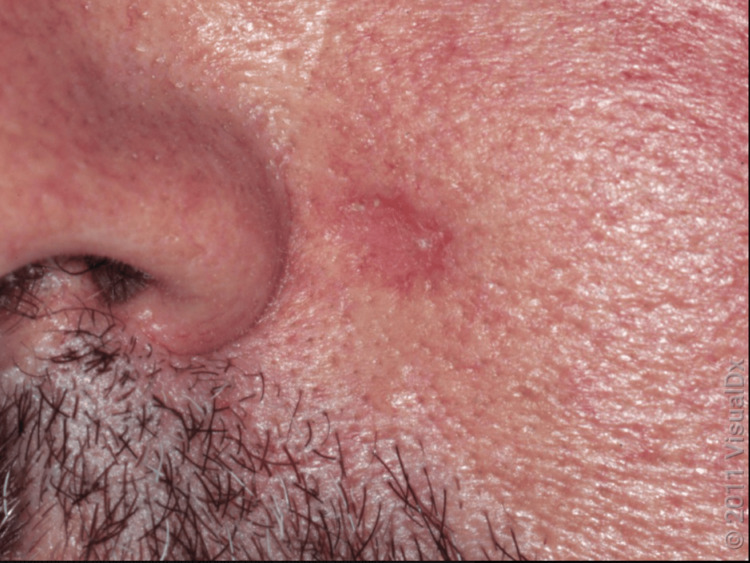
A close-up of a shiny telangiectatic nodule with scant overlying scale as seen in morpheaform BCC BCC: Basal cell carcinoma Image used with permission from VisualDx (www.visualdx.com) [4].

Basal cell carcinoma is the most prevalent form of skin cancer in the United States, affecting approximately 20% of Americans, with over 4.3 million new cases each year [2]. Though it is seldom life-threatening, BCC can lead to significant destruction and disfigurement of surrounding tissues if treatment is delayed or insufficient. Most BCCs develop from the basal cells of the epidermis, though a minority originate from the outer root sheath of the hair follicle [3]. The growth of the tumor can lead to ulceration, which may produce a rolled edge or rodent ulcer appearance. Often, patients report crusting and recurrent bleeding, prompting them to seek medical advice. Morpheaform BCC, which may appear as a scar-like plaque or a patch of morphea, is another variant of this condition (Figure 4).

**Figure 5:**
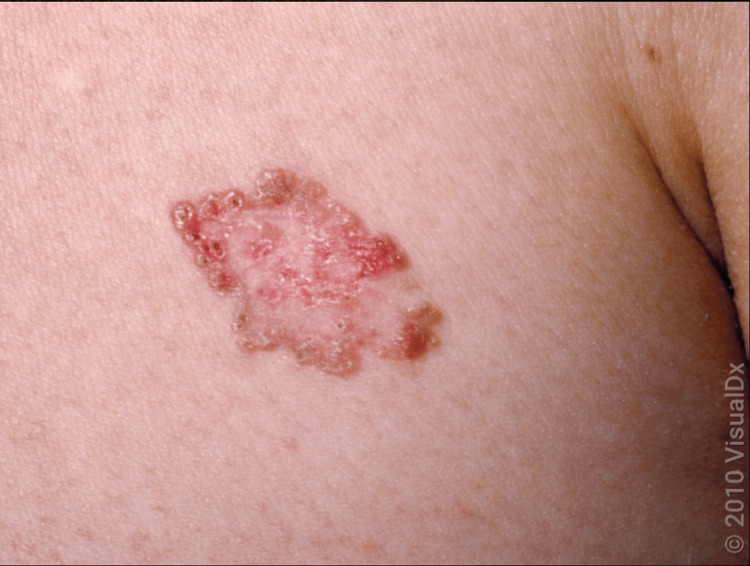
A close-up of a large annular plaque with a shiny, brownish, rolled border and a pink center with reddish papules and scant overlying scale. Image used with permission from VisualDx (www.visualdx.com) [5]

The lesion's surface often appears smooth but may include crusts with underlying erosions or ulcerations. Additionally, superficial BCC is characterized by faint, erythematous scaling plaques that gradually expand, typically found on the trunk and proximal limbs (Figure 5).

**Figure 6:**
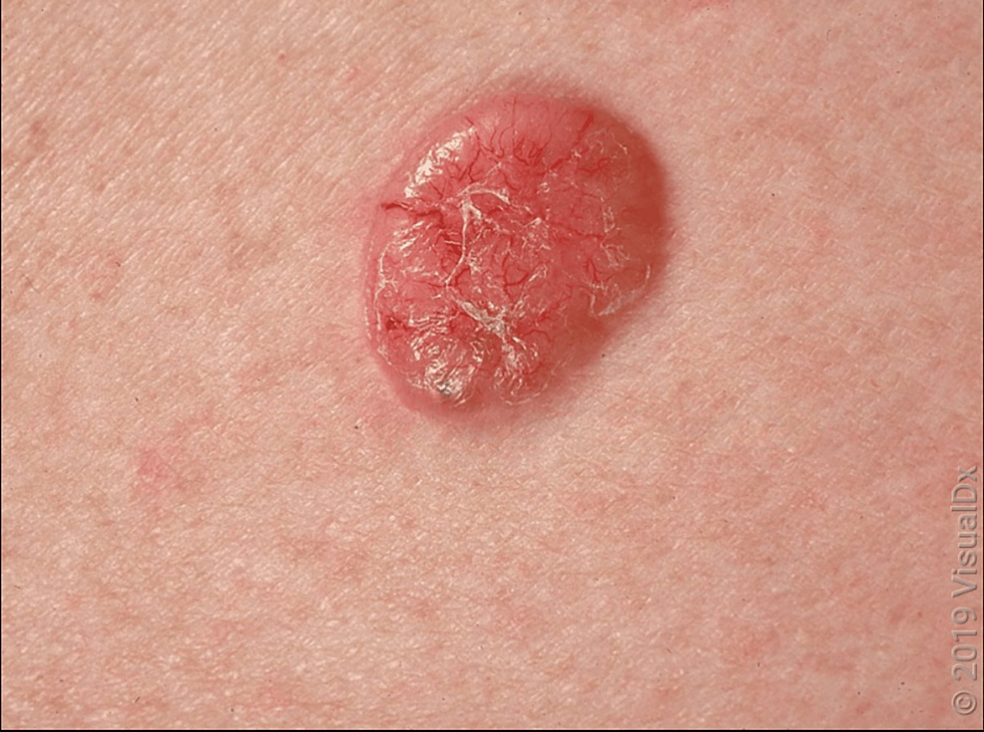
A close-up of a shiny telangiectatic nodule with scant overlying scale. Image used with permission from VisualDx (www.visualdx.com) [7]

Basal cell carcinoma can also present as a small, slowly growing, pearly nodule, often with tortious telangiectatic vessels on its surface, rolled borders, and a central crust. This kind of BCC is known as nodular BCC (Figure 6). This is the most common variant of BCC [6].

**Figure 7:**
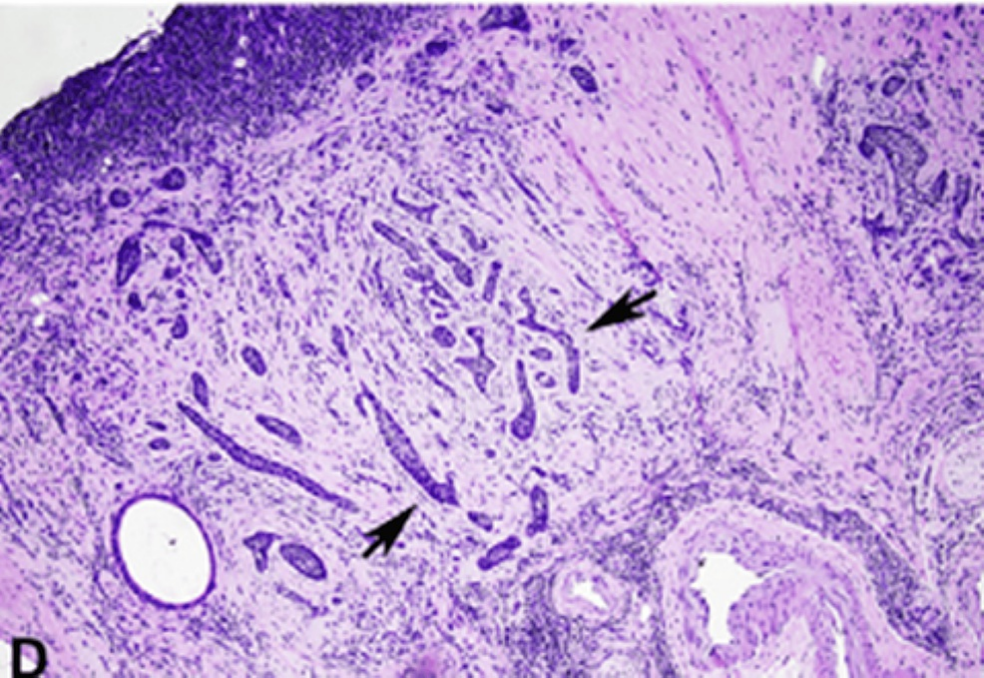
Shave biopsy showing infiltrative BCC. Arrows point to thin nests and cords of basaloid cells, some of which may have an irregular outline with pointed spiky projections, interspersed and infiltrating between dermal collagen bundles. BCC: Basal cell carcinoma Image reused from *Accuracy of biopsy sampling for subtyping basal cell carcinoma *by Andrea L et al.* *with permission from Elsevier [11].

Infiltrative BCC, known for its potential aggression and invasiveness, often features indistinct borders, and the lesions may appear subtle, which can delay diagnosis and treatment. These tumors tend to be more extensive than clinically expected, as demonstrated by our case in Figure 1 [8]. Diagnostic confirmation of BCC and its specific histological subtype requires a skin biopsy, including a shave, punch, or excisional biopsy, including dermal tissue, to distinguish between superficial and more invasive types [9]. Histologically, BCC biopsies reveal irregular thin nests and cords of basaloid cells, which sometimes display spiky, irregular projections intermingled with the skin's collagen fibers (Figure 7) [10]. Our patient was diagnosed with the infiltrative subtype of BCC.

The origins of BCC involve a complex interplay of genetic makeup, physical characteristics, and environmental exposure. A significant risk factor for BCC development is exposure to ultraviolet (UV) light, especially UVB rays, which are particularly intense for individuals who work outdoors [1]. Leisure activities in the sun and using tanning beds also contribute to the risk. Ultraviolet light therapy is another known precursor to BCC development. The UVB light can create mutagenic changes in DNA, such as forming cyclobutane pyrimidine dimers, impacting critical genes like p53 that control the cell cycle [3]. There is a noted recurrence rate of 12%, 10 years post-standard surgical removal. Individuals with a history of BCC are at a significantly increased risk, i.e., up to 10 times more likely to develop additional skin cancers, including non-melanoma skin cancers (NMSC) and melanoma [8]. Regular skin checks every six to 12 months for the first two years after a BCC diagnosis and annually thereafter are recommended if no further skin cancers are detected. Our case involves a road construction worker who frequently spent extended periods in the sun without protective sunscreen, likely exacerbating his risk for BCC.

About 20% of BCC cases develop on body parts not typically exposed to the sun. These instances might be linked to other risk factors such as ionizing radiation, exposure to arsenic, immunosuppression, and genetic factors like xeroderma pigmentosum, basal cell nevus syndrome, Bazex-Dupre-Christol syndrome, and Rombo syndrome. In women, smoking has been identified as a risk factor [8].

Men are generally more susceptible to BCC than women. The incidence is higher in regions with intense UV radiation due to geographical positioning near the poles or the equator. A previous history of squamous cell carcinoma, or BCC, strongly predicts future BCC risk. The median age at diagnosis is around 68 years. While BCC-related deaths are rare and typically occur in immunocompromised individuals, metastasis, when it happens, might affect regional lymph nodes, bones, lungs, and skin.

The primary aim of treatment for BCC is to remove the tumor altogether to prevent future recurrence, address any functional impairments caused by cancer, and achieve the best possible cosmetic outcomes, as BCC frequently affects the face. Surgical treatments, including excision, electrodesiccation and curettage, cryosurgery, and Mohs micrographic surgery, are preferred for localized lesions and boast high five-year cure rates, often exceeding 95%. Basal cell carcinoma seldom advances to severe stages, so systemic chemotherapy is rarely employed. Instead, advanced cases may be managed with targeted therapies or immunotherapy [11].

In most BCCs, the cells have mutations in genes that are part of a cell signaling pathway called hedgehog. The hedgehog pathway is crucial for developing the embryo and the fetus and continues to maintain importance in some adult cells. This pathway is kicked into overdrive in BCC, leading to the basal cells' hyperproliferation. Therefore, hedgehog pathway inhibitors target a protein in this pathway, inhibiting the basal cells' hyperproliferation. Examples of these drugs include vismodegib (Erivedge) and sonidegib (Odomzo). They are sold as capsules and are taken once a day. These medications are critical in treating BCCs that have spread to those that have returned after surgery or radiation therapy or that cannot be treated with surgery or radiation, such as the patient highlighted in this case report. These targeted drugs can help shrink the tumors or slow their growth. Some common side effects include muscle spasms, joint pain, hair loss, fatigue, dysgeusia, poor appetite, weight loss, nausea and vomiting, itchy skin, diarrhea, and constipation. In women, these drugs can cause cessation of menstrual periods. These drugs should be avoided in pregnant patients as they are teratogenic. Women of reproductive age taking these medications should use reliable birth control during treatment and sometimes following the completion of therapy [1].

## Conclusions

Patients with a history of BCC require ongoing and vigilant monitoring, especially those with multiple or high-risk tumors. This case report serves as a gentle reminder for healthcare providers to actively promote skin cancer prevention strategies among their patients. Encouraging habits like avoiding peak sun exposure, wearing protective clothing, and using sunscreen can significantly reduce the risk of BCC development.

Fortunately, the outlook for BCC remains positive, with excellent cure rates achieved through surgical and other treatment methods. While the likelihood of metastasis is low, lesions situated on the face or penetrating deeper tissues may have a less favorable prognosis. Regular follow-up appointments, at least annually, are essential post-diagnosis and treatment, as the risk of BCC recurrence within three years after an initial diagnosis is notable at 44%. By maintaining consistent surveillance and emphasizing preventive measures, we can continue to support our patients in their journey toward skin health and well-being.
